# Author Correction: Motion estimation for large displacements and deformations

**DOI:** 10.1038/s41598-022-26246-3

**Published:** 2022-12-14

**Authors:** Qiao Chen, Charalambos Poullis

**Affiliations:** Immersive and Creative Technologies Lab, Concordia University, Montreal, QC Canada

Correction to: *Scientific Reports* https://doi.org/10.1038/s41598-022-21987-7, published online 16 November 2022

The original version of this Article contained errors in the legends of Figure 8 and 10.

Figure 8. “Density of matches. The first row (a,b) shows an example of the input image frames, copyright of Valcarier DRDC, (c) shows SIFT^41^ matches, (d) shows RootSIFT^28^ matches, (e,f) shows EpicFlow^10^ and HybridFlow results. © His Majesty the King in Right of Canada, as represented by the Minister of National Defence, 2022.”

now reads:

Figure 8. “Density of matches. The first row (a,b) shows an example of the input image frames, © His Majesty the King in Right of Canada, as represented by the Minister of National Defence, 2022., (c) shows SIFT^41^ matches, (d) shows RootSIFT^28^ matches, (e,f) shows EpicFlow^10^ and HybridFlow results.”

Figure 10. “(a,b) Are two consecutive large-scale aerial images of a downtown urban area with resolution 6600 × 44006600 × 4400, copyright of Valcarier DRDC. (c) HybridFlow is the only top-performing variational method that can handle high-resolution images. Deep learning techniques cannot be applied due to the fixed input size of the networks as explained in the text. (d) Image resampled from (a) using HybridFlow flows in (c) to form (b). (e) Reconstructed pointcloud using 320 images. © His Majesty the King in Right of Canada, as represented by the Minister of National Defence, 2022.”

now reads:

Figure 10. “(a,b) Are two consecutive large-scale aerial images of a downtown urban area with resolution 6600 × 4400, © His Majesty the King in Right of Canada, as represented by the Minister of National Defence, 2022. (c) HybridFlow is the only top-performing variational method that can handle high-resolution images. Deep learning techniques cannot be applied due to the fixed input size of the networks as explained in the text. (d) Image resampled from (a) using HybridFlow flows in (c) to form (b). (e) Reconstructed pointcloud using 320 images.

The original Article has been corrected.

